# Removal of Endotoxin from rAAV Samples Using a Simple Detergent-Based Protocol

**DOI:** 10.1016/j.omtm.2019.08.013

**Published:** 2019-09-06

**Authors:** Liudmyla Kondratova, Oleksandr Kondratov, Ragy Ragheb, Sergei Zolotukhin

**Affiliations:** 1 Department of Pediatrics, Division of Cellular and Molecular Therapy, University of Florida, Gainesville 32610, FL, USA; 2 Malvern Panalitycal, Westborough, MA, USA

## Abstract

Endotoxin is the most common contaminant found in protein samples. Even a small amount of endotoxin can induce strong allergic reaction and death of a host organism. Endotoxin is also often detected in recombinant adeno-associated virus (rAAV) stocks prepared in research laboratories using off-the-shelf reagents; purifying rAAV stocks from endotoxin using commercial reagents sometimes results in significant titer loss. The problem is exacerbated due to the recently expanded diversity of rAAV serotypes and capsid variants, which, due to their variable capsid surface charge, display differential affinity toward endotoxin. In this paper, we describe a simple universal protocol of purifying vector stocks irrespective of AAV serotype. The protocol is based on subjecting endotoxin-contaminated rAAV to mild detergent treatment, followed by repeated buffer-exchange washing and concentrating viral stock by low-speed centrifugation. Multiple assays were employed to test the physical and biological equivalency of the viral stocks before and after purification. The described protocol has been routinely utilized to purify vector stocks contaminated at levels as high as >1,000 endotoxin units (EU)/mL to produce viral vectors with practically undetectable levels of endotoxin (<2.5 EU/mL), with the titer’s recovery in the range of 50%–100%.

## Introduction

Endotoxin is a lipopolysaccharide (LPS) present in the outer membrane of Gram-negative bacteria. LPS consists of O-antigen (distal polysaccharide), a core oligosaccharide, and a lipid A. LPS plays a protective and structural role for bacteria, but it can cause a strong adaptive immune response in mammals by activating the release of pro-inflammatory mediators, such as tumor necrosis factor (TNF), interleukin (IL)-6, and IL-1. Cell activation by LPS triggers the clinical syndrome of Gram-negative sepsis that can lead to fever, hypotension, respiratory and renal failure, and intravascular disseminated coagulation. These reactions are induced upon intravenous injection of LPS at concentrations as low as 1 ng/mL, which is equivalent to about 10 LPS units (endotoxin units [EU]).^1^ Consequently, according to US Food and Drug Administration (FDA) guidelines, the levels of LPSs in intravenously injected biopharmaceuticals must not exceed 5 EU/1 kg body weight.^2^

LPS is a chemically stable molecule with a net negative charge; it is resistant to high pressure, extreme temperatures, and pH values. The molecular mass of LPS monomers varies from 10 to 20 kDa, which, in aqueous solutions, can form stable aggregates (micelles) with a diameter up to 0.1 μM. Divalent cations, such as Ca^2+^ and Mg^2+^, are known to stabilize the aggregated structure of LPS, whereas detergents help to break down micelles into smaller sub-units.^3^

Both basic (isoelectric points [pI] > 7) and acidic proteins (pI < 7) interact with LPS molecules through either electrostatic or hydrophobic interactions, respectively.^4^ These LPS/protein aggregates constitute a challenge for the pharmaceutical industry, requiring the implementation of protocols for the removal of LPS based on the unique molecular properties of both LPS and the target biopharmaceuticals.

Since bacteria can grow in nutrient-poor media, such as water, saline, and buffers, LPSs are found almost everywhere. In a laboratory setting, any buffer, commercial or prepared from powdered reagents, will contain some level of LPS. For example, PBS, commonly used in biological research, might contain up to 76 EU/mL if made from powder reagents or up to 5 EU/mL if purchased as a solution.^5^ Moreover, LPS tends to adhere to glass or plastic so that reusable lab-ware, shared equipment, and work surfaces utilized to handle bacteria can be a source of significant cross-contamination. More expensive, pyrogen-free certified H_2_O and other reagents must be used to reduce LPS below practical detection levels (<2.5 EU/mL).

Adeno-associated virus (AAV) is a virus incorporating single-stranded DNA (ssDNA) genome into protein shell and, as such, is susceptible to LPS contamination as well. In most cases, recombinant AAV (rAAV) is prepared after transfection of HEK293 cells with plasmid DNA isolated from *E. coli*. Outsourcing the production of LPS-free plasmid DNA to specialized commercial entities is a requisite but expensive requirement if Good Manufacturing Practice (GMP)-grade vectors are manufactured. Usually, however, the researchers themselves purify plasmid DNA using commercial kits and silica-based ion-exchange resins, expecting similar quality DNA. Unfortunately, due to the various reasons mentioned above, the outcome does not meet the expectations, leaving the researcher with few options to decontaminate an already concentrated and titered rAAV sample.

In this paper, we describe a simple universal protocol of purifying vector stocks irrespective of AAV serotype. The approach is based on the following facts: (1) LPS forms micelle aggregates and complexes with proteins, including AAV capsids, which are refractive to separation by differential ultrafiltration or ion-exchange chromatography; and (2) LPS could be removed by treating contaminated protein samples with a detergent.^3^ The described protocol includes treating contaminated stocks with mild detergent to dissociate LPS micelles, followed by repeated buffer-exchange washing and concentrating viral stock by low-speed centrifugation under conditions limiting re-aggregation. The treatment results in a high yield of the vector and does not modify physical properties or biological potency of the virus.

## Results

The analysis of the calculated pI mean values of capsids of all characterized AAV serotypes 1–12 showed their slightly acidic character (pI = 6.3).^6^ However, at the physiological pH range of pH 7–8, the clusters of positively charged basic amino acid residues provide sufficient substrate for electrostatic interactions of capsid proteins with negatively charged LPS molecules, either aggregated or monomers. This interaction is enhanced by micelle-stabilizing divalent cations (Ca^2+^ and Mg^2+^), thus presenting a technical challenge when attempting to separate LPS and rAAV.

### LPS Purification Workflow

The protocol was developed using AAV2, AAV2-DGEDF (AAV2-derived capsid mutant^7^), AAV5, AAV8, AAV9, AAVrh10, AAV-TT,^8^ and AAV-DJ.^9^ Some vector stocks were apparently contaminated during production; AAV9, AAVrh10, and AAV-TT,^8^ for the purpose of this project, were spiked with LPS to the levels >1,000 EU/mL. For decontamination, we used 200 μL of each sample. Purification was tested with two different detergents: Triton X-100 and sodium deoxycholate (Na-DC).

**Figure 1:**
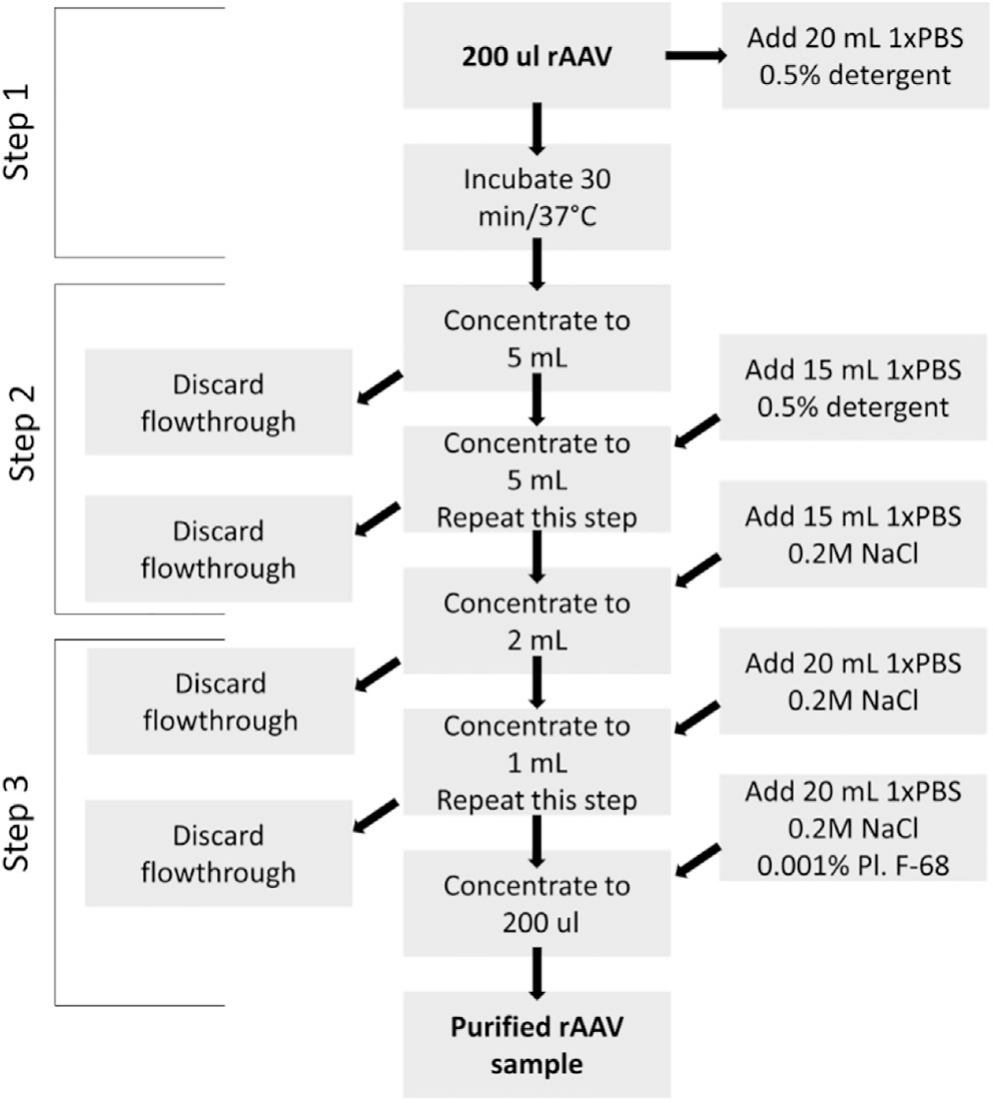
LPS Decontamination of rAAV Samples: Workflow

rAAV purification from LPS was performed in three distinct steps as shown in Figure 1.

#### Step 1

This step is aimed at dissociating LPS and AAV and also at reducing the size of LPS micelles in a low-salt buffer. The workflow includes dilution of rAAV sample 100-fold with PBS buffer (pre-warmed at 37°C) containing 0.5% detergent (Triton X-100 or Na-DC) and incubation of the mixture for 30 min at 37°C, the temperature promoting LPS dissociation. After the incubation, the sample is concentrated in the centrifugal spin filter (VS2042, 100,000 molecular weight cut-off [MWCO], Sartorius) at 2,000 × *g* to a final volume of about 5 mL. It is important to avoid concentrating sample to titers higher than 2 × 10^12^ vector genomes (vg)/mL (or 10^12^ for AAV2), because, at this stage, virus tends to aggregate and precipitate.

#### Step 2

This step of the workflow includes dilution of the sample with a pre-warmed (at 37°C) PBS-0.5% detergent and concentrating in the centrifugal filter unit using the same conditions as above. This step is repeated one more time to wash out the remaining LPS in a low-salt (PBS-0.5% detergent) buffer.

#### Step 3

This step includes replacing low-salt with high-salt AAV formulation PBS-0.2 M NaCl and washing the traces of detergent. Once the buffer composition is changed to a higher ionic strength, it is now safe to concentrate the virus down to its original volume and titer. Finally, to recover virus quantitatively, the buffer for the final washing step consists of 1× PBS-0.2 M NaCl-0.001% Pluronic F-68. The decontaminated virus is sterilized by passing through a 0.22-μm filter, and it is further used for quantitative and qualitative assays.

The described method helps to reduce LPS from about 1,000 EU/mL to practically undetectable levels of less than 2.5 EU/mL, regardless of the detergent used (Table 1).

**Table 1:** Endotoxin Levels and AAV Titer Recoveries

Sample	LPS (EU/mL)		Detergent	Viral Particles (vg)		Recovery (%)
	Before	After		Before	After	
AAV2	297	<2.5	Na-DC	3.6 × 10^11^	2.4 × 10^11^	66
AAV2	297	<2.5	Triton X-100	3.6 × 10^11^	2.8 × 10^11^	68
AAV2-DGEDF	193	<2.5	Na-DC	1.9 × 10^12^	1.09 × 10^12^	57
AAV2-DGEDF	193	<2.5	Triton X-100	1.9 × 10^12^	1.12 × 10^12^	59
AAV5	9	<2.5	Na-DC	9 × 10^12^	6.7 × 10^12^	74
AAV5	9	<2.5	Triton X-100	9 × 10^12^	8.7 × 10^12^	96
AAV9	10	<2.5	Na-DC	3 × 10^12^	1.5 × 10^12^	50
AAV9	10	<2.5	Triton X-100	6.4 × 10^11^	5.7 × 10^11^	89
AAV-rh10	>1,000	<2.5	Triton X-100	5 × 10^12^	4.6 × 10^12^	92
AAV2-TT^8^	>1,000	<2.5	Triton X-100	5 × 10^12^	3.4 × 10^12^	68
AAV-DJ^9^	60	<2.5	Triton X-100	3.7 × 10^12^	5.1 × 10^12^	73
AAV-Mut1	>1,000	<2.5	Triton X-100	4.2 × 10^12^	2.7 × 10^12^	64
AAV-Mut2	>1,000	<2.5	Triton X-100	5.9 × 10^12^	3.3 × 10^12^	54
AAV-Mut3	>1,000	<2.5	Triton X-100	4.7 × 10^12^	3.6 × 10^12^	78

### Virus Recovery after LPS Decontamination

The recovery of the virus titers after LPS purification was assayed by qPCR. Notably, the titers’ recoveries varied from 96% to 50%, depending on the AAV serotype and the detergent used during steps 1 and 2 (Table 2). Serotypes AAV2 and derivative thereof, such as rAAV2-DGEDF, were most susceptible to the loss, regardless of the type of detergent used. Meanwhile, rAAV5 and rAAV9 showed titers’ recoveries up to 96%, which were also detergent dependent. The main reason for titer loss is the aggregation during step 2 of the purification protocol when LPS is being removed in a low-salt buffer and the virus is concentrated. The titer recovery is higher for serotypes that are less likely to aggregate. Overall, Triton X-100 demonstrated better performance, and it is recommended for the final version of the protocol.

**Table 2:** Detergent Residues in Triton X-100-Treated Samples Assayed by 280-nm Absorbance

Sample	Titer (vg/mL)	Triton X-100 (%)
AAV2	10^12^	0.006
AAV2-DGEDF	5.6 × 10^12^	0.005
AAV5	2.5 × 10^13^	0.019^a^
AAV9	2.3 × 10^12^	0.005

a Higher absorption at 280 nm is attributed to higher capsid protein concentration of AAV5 in this particular sample.

### Characterization of Detergent Residuals

Triton X-100 was reported as having a toxic effect on cells at concentrations of 0.15 mM, which corresponds to 0.009% in the solution.^10^ A simple calculation of Triton X-100 dilution factor following the protocol’s workflow would result in 0.00003% final concentration. However, at higher concentrations, detergent monomers aggregate into structures called micelles. A micelle is a thermodynamically stable colloidal aggregate of detergent monomers wherein the nonpolar ends are sequestered inward, avoiding exposure to water, and the polar ends are oriented outward, in contact with the water. The formation of micelles alters the flow rate of the detergent through the centrifugation filter device, requiring experimental testing of detergent residuals.

The assay CMC-535 has been used to determine detergent concentration during the purification procedure. As the assay includes a dye sensitive to phosphates, the measurements cannot be performed in PBS buffer. Therefore, for the purpose of detergent quantification, we substituted Lactated Ringer’s solution for PBS buffer in the purification protocol. As a result, we were able to perform measurements only for Triton X-100, as 0.5% Na-DC in Lactated Ringer’s solution forms a viscous gel-like solution.

Following the protocol workflow and prior to Pluronic F-68 addition, the detergent residuals in the final virus stock contained 0.008% Triton X-100. To exclude the possibility of differential performance of the detergent in PBS buffer versus Lactated Ringer’s, we used an additional assay based on Triton X-100 characteristic absorption at 280 nm and a standard dilution curve of Triton X-100 in PBS-0.2 M NaCl ranging from 0.1% to 0.0016%. The measured Triton X-100 concentrations in the purified samples in the presence of PBS buffer were found to be 0.005%–0.006% for the viral stocks with the titers of about 10^12^ vg/mL, in close correlation with the result of CMC-535 assay (Table 2). Notably, in one sample of AAV5 with the higher titer of 2.5 × 10^13^, the absorption of AAV capsid proteins at 280 nm rendered the overall absorption somewhat higher (0.019). Therefore, the residual of Triton X-100 in the purified virus samples can be considered safe to be administered *in vivo*.

### Characterization of AAV Biological Potency

**Figure 2:**
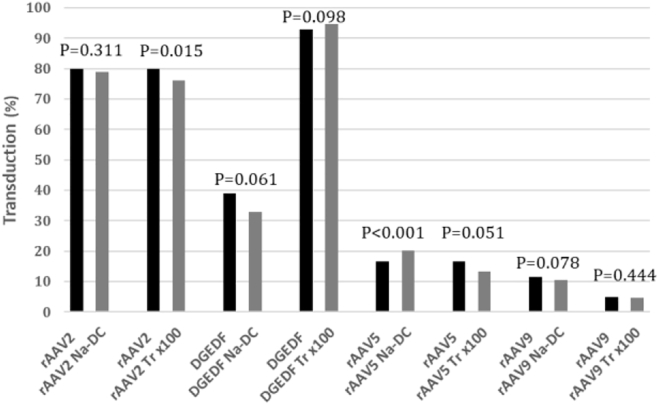
Biological Potency of rAAV before and after LPS Decontamination P-values quantified by unpaired two-sample T-test. Each bars’ pair represents transduction efficiency of HeLa C12 cells (% of cells transduced at the same MOI), before and after decontamination using different detergents (sodium deoxycholate or Triton X-100). Black and gray bars represent transduction efficiencies before and after LPS decontamination, respectively.

Treatment AAV with 0.5% detergent at 37°C for an extended period of time raised the possibility of capsid inactivation. To this end, we assessed transduction efficiency of rAAV samples *in vitro* before and after the LPS decontamination procedure. Overall, after detergent-based purification from LPS, the transduction efficiency remained unchanged for the majority of the samples tested (p < 0.05) (Figure 2). However, we documented a reduction in biological potency for rAAV2 after Triton X-100 treatment by 5%–20%, depending on the MOI assayed (p = 0.015). On the other hand, Na-DC-treated rAAV5 demonstrated a 20% increase in infectivity (p < 0.001). Therefore, we conclude that detergent treatment, in most cases, does not affect the biological potency of the virus.

### Capsid Stability

We characterized rAAV capsids by three independent assays: (1) Sodium dodecyl sulfate–polyacrylamide gel electrophoresis (SDS-PAGE) followed by silver staining, (2) capsid thermostability (also known as AAV-ID^11^), and (3) electron microscopy.

#### SDS-PAAG

**Figure 3:**
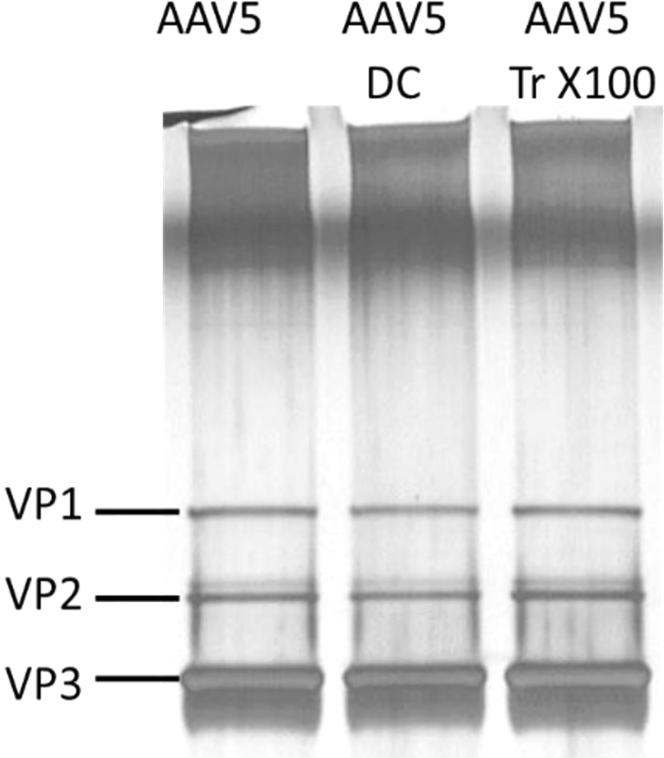
rAAV Capsid Protein Ratio and Thermostability following LPS Decontamination Procedure Decontamination procedure doesn’t affect capsid protein ratio, as judged by silver-stained SDS-protein gel of AAV5.

Analysis of viral capsid mobility in a gel allows assessing changes in VP1, VP2, and VP3 capsid protein composition and potential degradation. Representative samples of AAV5 (5 × 10^10^ vg/well) were analyzed before and after LPS decontamination. As shown in Figure 3, the ratios, the content, and molecular weights of capsid proteins VP1, VP2, and VP3 are consistent among the three samples analyzed. Therefore, the protein composition of capsids is unchanged after treating with either Na-DC or Triton X-100.

#### Thermostability of AAV Capsid

**Figure 4:**
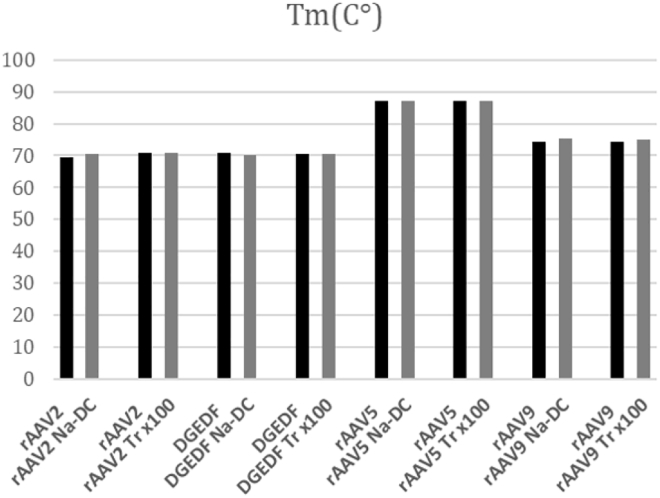
Thermostability of the Capsids of Different AAV Serotypes before and after LPS Removal

Thermostability assay, so-called AAV-ID, is based on the ability of SYPRO Orange dye to become fluorescent upon binding to hydrophobic amino acid residues.^11^, ^12^ With an increase in temperature, more hydrophobic residues in the AAV capsid structure are externalized and fluorescence increases. The melting temperature (Tm) is a temperature at which the derivative fluorescence signal reaches its maximum value. The Tm obtained for four sets of rAAV samples (Figure 4) shows a 0.5°C increase for AAV9 after Triton X-100 treatment and a 1°C increase for Na-DC-treated AAV2 and AAV9. If anything, the decontamination procedure increased the thermostability of AAV capsids.

#### Electron Microscopy

**Figure 5:**
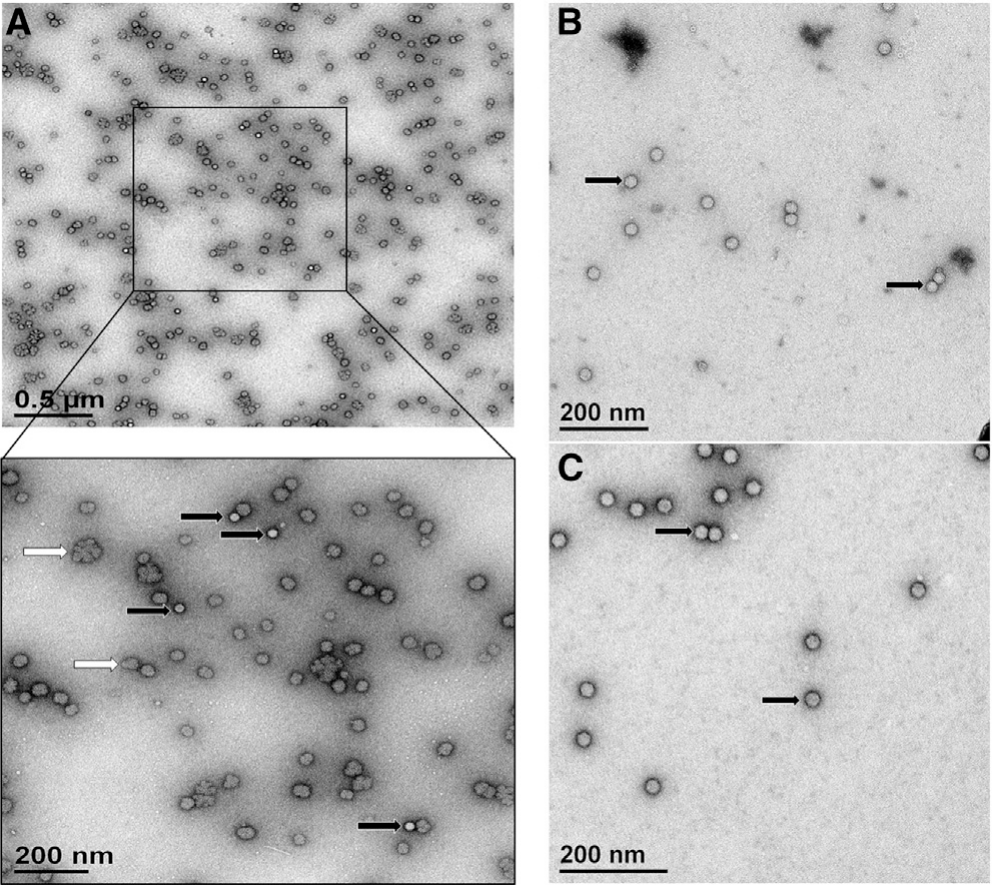
Analysis of rAAV2 Vector Spiked with LPS and after LPS Decontamination via Negative-Stain Transmission Electron Microscopy (A–C) rAAV2 sample spiked with LPS (25 μg/mL) (A), rAAV2 preparation purified from LPS using Na-DC (B), and rAAV2 preparation purified from LPS using Triton X-100 (C). The black arrows indicate rAAV particles; the white arrows point to LPS aggregates.

To visualize endotoxin and its potential interaction with the virus, the purified AAV2 sample was spiked with LPS from *E. coli* O111:B4 at the concentration of 25 μg/mL (corresponding to >1,000 EU/mL) and subjected to negative-stain electron microscopy (Figure 5A). As seen in the inset of Figure 5A, LPS micelles form heterogeneous structures bigger in size than AAV capsids, sometimes forming bigger still snowflake-like shapes. AAV capsids are often found in close contact with LPS micelles, suggesting their physical interactions. After decontamination to the levels of <5 EU/mL with the Na-DC protocol (Figure 5B), or with Triton X-100 (Figure 5C), viral capsids remained intact. Overall, capsid analysis showed that LPS decontamination using the detergent-based approach does not change capsid morphology.

### Assessing AAV Particle Aggregation

The dissociation of LPS micelles induced by detergent is enhanced by the reduction of the ionic strength of the buffer. As a result, the conditions of step 2 of the decontamination workflow in a low-salt (1× PBS) buffer increase the risk of a reduced virus recovery due to its aggregation. Consequently, one of our goals was to identify the maximal titer that would not initiate virus aggregation during purification. The process has been monitored by a light-scattering device measuring the average size of major particle population in a solution. As the diameter of an AAV particle is about 25 nm, the higher average number would indicate the particle aggregation.

**Figure 6:**
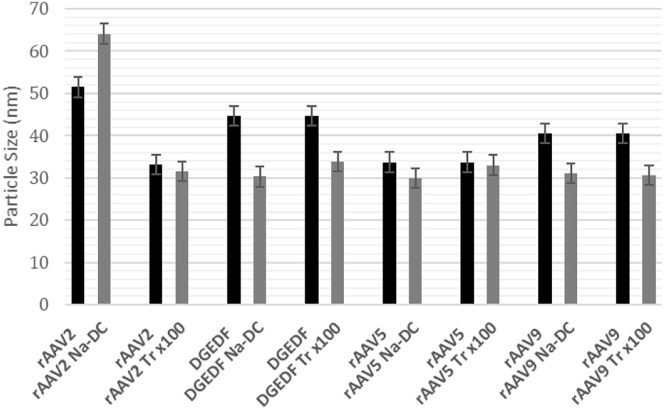
Particle Size of rAAV before and after Detergent-Based Purification Bars represent standard error.

Figure 6 shows an average particle size before and after purification. We observed that the average particle size remained constant or even decreased for AAV-DGEDF, AAV5, and AAV9. At the same time, AAV2 showed an aggregation trend after Na-DC treatment (the average particle size increased from 51 to 63 nm) while not changing the average particle size after Triton X-100 treatment. Empirically, we have determined that during washing in 1× PBS buffer (step 2), it was critical not to concentrate AAV samples to titers higher than 2 × 10^12^ vg/mL (or 10^12^ for AAV2). The safe final titer of the virus concentrated in 1× PBS-0.2 M NaCl (step 3) could be increased up to 10^13^ vg/mL (or 5 × 10^12^ vg/mL for AAV2). One has to keep in mind though that AAV vectors for this project were purified using double sequential iodixanol purification protocol and, thus, were essentially free of empty capsids. The potential contamination with empty AAV particles should be taken into account to determine the safe titers during purification.

## Discussion

According to the FDA requirements, the levels of LPS in intravenously (IV)-injected drugs should not exceed 5 EU/kg. Because the majority of steps during rAAV production are carried out outside the biosafety cabinet, the risk of viral stock contamination with environmental LPS is significant. Due to the complexity of the structural features of AAV capsids, the multitude of available serotypes, and the chemical nature of LPS forming intricate micelle structures while interacting with AAV, the available commercial methods of LPS removal using affinity chromatography, in our hands, often resulted in poor (<10%) virus recoveries. Hereby, we describe a protocol that reduces LPS level in rAAV preparations to practically undetectable levels (<2.5 EU/mL), while maintaining relatively high virus recoveries. It is designed primarily for academic laboratories and, in its current form, is not intended for large-scale virus production in an industry setting. The described method allows for a simple decontamination of rAAV of different clades and serotypes using off-the-shelf inexpensive reagents. Importantly, the protocol does not alter structural properties of AAV and its biological potency.

It has been established earlier that detergents dissociate LPS micelles; however, the optimal concentrations of detergents to decontaminate AAV while maintaining its structure and functions were not known. We studied the effects of nonionic detergent Triton X-100 and a mild ionic detergent, sodium deoxycholate (a sodium salt of a bile acid), on different physical and biological properties of AAV. The inclusion of a detergent into the protocol as an active agent necessitates its subsequent removal from the purified virus stock. Detergent monomers solubilize cell membrane proteins by partitioning into the membrane bilayer. With increasing amounts of detergents, membranes undergo various stages of solubilization; therefore, detergents should be avoided at high concentrations in samples administered *in vivo*. Moreover, detergents often contain trace impurities from their manufacture. Some of these impurities, especially peroxides that are found in most nonionic detergents, will inhibit protein activity.

Therefore, we’ve extensively tested decontaminated virus stocks for the detergent residuals and established their level to be below toxic. Notably, detergents were shown to decrease the ability of Limulus Amebocyte Lysate (LAL)-based assay (an assay used in this project) to detect LPS,^13^ due to the so-called masking effect. A masked LPS is a monomer surrounded by the molecules of a detergent. Because of this masking effect, LAL assay might show a false-negative result for the tested sample if the concentration of Triton X-100 exceeds 0.05%. In our decontaminated stock, the Triton X-100 concentrations were 10 times lower. To avoid a false-negative result in our approach, we also calibrated the LAL assay in a way that the LPS levels in final samples were not higher than the negative control. Interestingly, the biological activity of masked LPS is reported to be ten times lower compared to the aggregated LPS, since monomers are considered to be biologically less active.^13^ Data for the masking activity of Na-DC are not available.

The melting temperature of the capsid is considered to be an important benchmark of the capsid structural integrity and serotype identity.^12^ The documented slight changes in Tm in some decontaminated vector stocks did not exceed 1°C and mostly occurred for Na-DC-treated samples. This little change can be attributed to the ability of this detergent to bind to SYPRO Orange dye, which was used for measuring rAAV thermostability.^14^ Among Triton X-100-treated samples, a 0.5°C increase in Tm was also shown for rAAV9, which is consistent with the variability in Tm for rAAV9 shown earlier to be about 0.8°C.^11^

LPS aggregates exist in many forms (micelles, cubic/hexagonal aggregates, and snowflake-like), all of which are dependent on buffer composition and LPS concentration.^15^, ^16^ The LPS micelles at a concentration of 25 μg/mL in some published negative staining electron micrographs appear to be tubular.^17^, ^18^ In this paper, we were able to identify only cubic and snowflake aggregates in the spiked samples that were formed during sample formulation. In regular purified rAAV samples, the storage buffer contains high concentration of Na^+^, phosphates, and Ca^2+^; all of them might play a role in the aggregation and stabilization of aggregates. However, the levels of LPS contamination in such samples (usually ∼200–300 EU/mL) are below the detection levels by negative staining electron microscopy. We did, however, visualize tubular aggregates by limited dilutions of commercial LPS stock solution in LPS low-salt buffer (data not shown). Apparently, in high-salt buffers, which are used for storing rAAV, the majority of LPS contaminants exist in cubic or snowflake aggregate form.

The biological potency of a virus is a critical characteristic of an rAAV vector. For the majority of the tested samples, the decontamination procedure didn’t change *in vitro* infectivity. However, the assay documented a reduction for rAAV2 infectivity after Triton X-100 treatment by 5%–20% (depending on the assay and MOI) and, surprisingly, an increase for rAAV5 after Na-DC treatment by the same degree. It was earlier shown that cationic polymers and lipids can alter the infectivity of rAAV.^19^, ^20^, ^21^, ^22^ It is conceivable that more than a 20% transduction increase for rAAV5 after Na-DC treatment is caused by detergent residuals in the sample, especially given that Na-DC is known to form polymers.^23^

In conclusion, we have developed a simple protocol that reduces LPS level in rAAV preparations to undetectable levels (<2.5 EU/mL) while maintaining relatively high virus recoveries. The described method allows for the decontamination of rAAV of different clades and serotypes using off-the-shelf inexpensive reagents. The treatment does not alter structural properties of rAAV and its biological potency.

## Materials and Methods

### AAV Preparation

rAAVs used in the project were produced by standard polyethylenimine (PEI)-mediated plasmid co-transfection in HEK293 cells. The virus was purified using double sequential iodixanol gradient protocol, as described by Crosson et al.^24^ Purified viruses were stored in 1× PBS-0.2 M NaCl-0.001 Pluronic F-68.

### rAAV Titering

rAAV titer was determined by qPCR using CMV promoter primers, qPCR mix (Applied Biosystems, A25742), and a Bio-Rad CFX Connect RealTime System.

### LPS Detection

LPS levels were detected using LAL assay (GenScript, L00350) with 0.1–1 EU/mL linear range. Kits of separate lots have distinct calibration curves that could be different from one lot to another. To stay within the linear range of the assay, all samples were diluted 25, 100, and 1,000 times in LPS-free water provided with the kit.

### Detergent Quantification

The level of residual detergent in the samples after purification was measured only for Triton X-100, as we were unable to find suitable methods for Na-DC identification in a sample. Levels of Triton X-100 contamination was detected by two different methods: CMC-535 Detergent Assay (Bioscience) and measuring the sample absorption at 280 nm.

### CMC-535 Detergent Assay

Although all LPS purification protocols were carried in PBS-based buffer, for detergent assay, all steps were repeated with Lactated Ringer’s solution because the assay is limited to the buffers containing no phosphates. Also, we were unable to measure Na-DC level after LPS purification due to its polymerization at low pH.^25^ For each detergent, the assay requires its own standard calibration curve; therefore, an aliquot of the sample was assayed prior to Pluronic F-68 addition.

### Absorption at 280-nm Wavelength

Another method for assaying detergent is absorption of the sample at 280 nm, as described by London et al.^26^ In this case, the assay was performed on the final sample containing Pluronic F-68, which does not interfere with the measurements. To establish a standard curve, seven serial dilutions of the PBS-0.2 M NaCl buffer were prepared containing dilutions of Triton X-100 ranging from 0.1% to 0.0016%. The absorbance of the viral capsid protein at titers of 10^12^ vg/mL was found to be negligible to interfere with the calculations.

### *In Vitro* Transduction Assay

HeLa C12 cells^27^ were cultured in 24-well tissue culture dishes in high glucose DMEM, supplemented with 10% fetal bovine serum (FBS) and Antibiotic-Antimycotic Solution (Cell Applications). rAAVs incorporating mApple^28^ or Antares^29^ reporter genes were used to infect C12 cells at ∼80% confluency with different MOIs (depending on serotype) and incubated 1 h. After incubation, cells were infected with Ad5 (MOI of 5) and incubated for another 48 h in a 5% CO_2_ humidified environment at 37°C. After incubation, cells were harvested and analyzed by fluorescence-activated cell sorting (FACS).

At least three wells were infected with each virus. To quantify statistical significance between each pair of viruses (original virus versus sodium deoxycholate purified and original virus versus Triton X-100 purified), the two-sample t test was performed using JASP software.

### Capsid Thermostability

Capsid thermostability was measured as described by Pacouret et al.^11^ using differential scanning fluorimetry (DSF).

### Electron Microscopy

Contaminated with LPS (Sigma, L4391) and purified rAAV were negative stained with uranyl acetate 1% and examined using FEI Spirit Transmission electron microscopy at 120 kV.

### Silver Staining

Silver staining of samples before and after purification was performed with Pierce Silver Stain Kit (Thermo Scientific) and precast gels (Bio-Rad). rAAVs were loaded at 5 × 10^10^ vg/well.

### Particle Size

Particle size was measured through dynamic light scattering (DLS) using a Malvern Panalytical Zetasizer Ultra with ZS Explorer software (version 1.1.0.656). Each sample was measured 5 times at a non-invasive backscatter angle in a 10 × 10 disposable cuvette.

## Author Contributions

Conceptualization, L.K. and O.K.; Methodology, L.K., O.K., and S.Z.; Investigation, L.K., O.K., and R.R.; Writing – Original Draft, L.K.; Writing – Review & Editing, S.Z.; Supervision, S.Z.
